# Cancerous Arrhythmia: Metastatic Cervical Cancer to the Heart Complicated by Ventricular Tachycardia

**DOI:** 10.1016/j.jaccas.2026.108560

**Published:** 2026-06-02

**Authors:** Sarah Noorani, Kevin S. Tang, Akash Patel, Daniel Tran, Amin Sabet

**Affiliations:** a Department of Medicine, University of California-Irvine Medical Center, Orange, California, USA; b Division of Cardiology, UCSF, San Francisco, California, USA; c Mary & Steve Wen Cardiovascular Division, University of California-Irvine Health, Irvine, California, USA; d Department of Pathology, University of California-Irvine Medical Center, Orange, California, USA

**Keywords:** cardiac metastasis, cervical cancer metastasis, malignant cardiac mass, ventricular arrhythmia

## Abstract

**Background:**

Cardiac metastasis from cervical cancer presenting with ventricular arrhythmias is an exceedingly rare phenomenon that requires a multidisciplinary approach for management and prognostication.

**Case Summary:**

A woman with cervical squamous cell carcinoma presented with nonsustained ventricular tachycardia. Imaging revealed a 4-cm right ventricular mass with biopsy consistent with metastatic cervical squamous cell carcinoma. The patient was a poor surgical candidate and was managed with chemotherapy and ambulatory cardiac monitoring with close outpatient follow-up.

**Discussion:**

Cardiac metastasis is a rare occurrence that requires consideration of surgical candidacy, appropriate cytotoxic therapy, and management of complications. Ventricular arrhythmias are infrequently described but should be first managed with antiarrhythmic drugs, although sparse reports exist of radiofrequency ablation.

**Take-Home Messages:**

Dangerous ventricular arrhythmias are a rarely described complication of cardiac metastasis from cervical cancer. Optimal management is poorly defined but should involve a multidisciplinary approach and consideration of both surgical and medical therapies.

## Visual Summary

**Figure:**
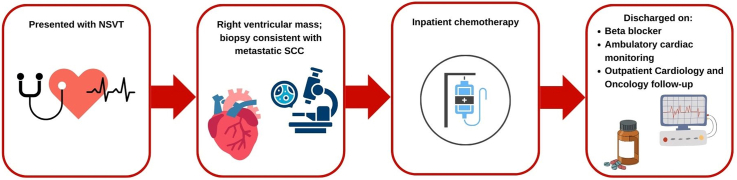

**Visual Summary:**
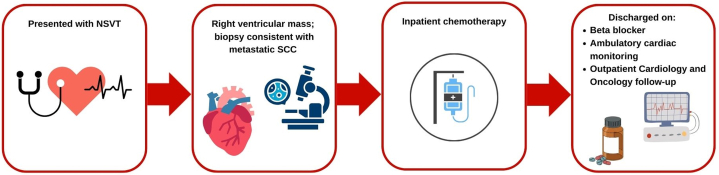
Management of Cervical Squamous Cell Carcinoma with Cardiac Metastasis Complicated by Ventricular Tachycardia Patient presented with ventricular tachycardia due to right ventricular metastasis. Management includes surgical evaluation, chemotherapy, and medications for arrhythmia and symptom management. NSVT = nonsustained ventricular tachycardia; SCC = squamous cell carcinoma.

## History of Presentation

A 60-year-old woman with stage III cervical squamous cell carcinoma status-post 2 cycles of chemotherapy presented with palpitations. On presentation her vitals were normal and physical exam was unremarkable.

**Take-Home Messages**

• Dangerous ventricular arrhythmias are a rarely described complication of cardiac metastasis from cervical cancer.
• Optimal management is poorly defined but should involve a multidisciplinary approach and consideration of both surgical and medical therapies.

## Past Medical History

The patient's past medical history included type 2 diabetes mellitus, hypertension, and right hydronephrosis secondary to tumor burden status-post percutaneous nephrostomy tube placement. Before presentation, she had undergone 2 cycles of chemotherapy with carboplatin, paclitaxel, and pembrolizumab.

## Differential Diagnosis

The differential diagnosis included cardiac metastasis, chemotherapy-induced cardiotoxicity, electrolyte abnormalities, and structural heart disease.

## Investigations

The electrocardiogram on admission revealed sinus rhythm with nonspecific T-wave changes. She was placed on cardiac monitoring that revealed premature ventricular contractions and episodes of nonsustained ventricular tachycardia (NSVT) with the longest episode lasting 3 beats (Figure 1). The patient was asymptomatic and hemodynamically stable during these episodes. She was started on metoprolol succinate 50 mg daily to which she responded well.

**Figure 1:**
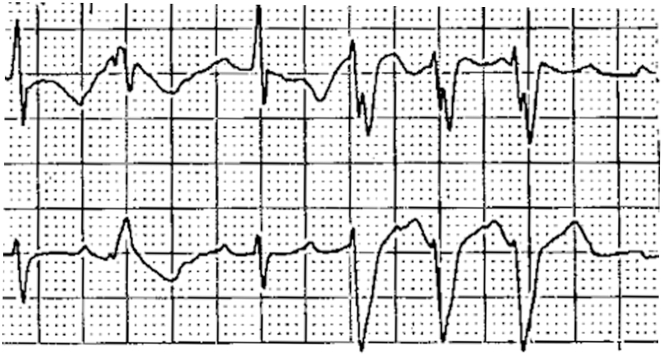
Electrocardiogram Revealing Ventricular Arrhythmia Sinus rhythm followed by 3 beats of ventricular tachycardia.

Given the patient's new arrhythmia, she underwent a transthoracic echocardiogram that demonstrated normal right and left ventricular systolic and diastolic function. However, it revealed a right ventricular mass that measured 4 × 2 cm. Given her history of malignancy, this was most concerning for cardiac metastasis. She underwent cardiac magnetic resonance (CMR) imaging that showed an apical right ventricular mass measuring 5.1 × 3.7 × 3.5 cm with central necrosis (Figures 2 and 3B). The right ventricle was normal in size and had a minimally depressed ejection fraction of 50%. The imaging demonstrated focal hypocontractility of the right ventricular septum and apical walls at the mid and apical segments due to the presence of the right ventricular mass, and there was no evidence of myocarditis. The patient also obtained a positron emission tomography (PET)-computed tomography (CT) scan that demonstrated the cardiac mass measuring 2.9 × 4.4 × 3.1 cm with maximum standardized uptake value of 7.0 (Figure 3A). The PET-CT also noted a reduction in size of the cervical mass indicative of partial response to chemotherapy.

**Figure 2:**
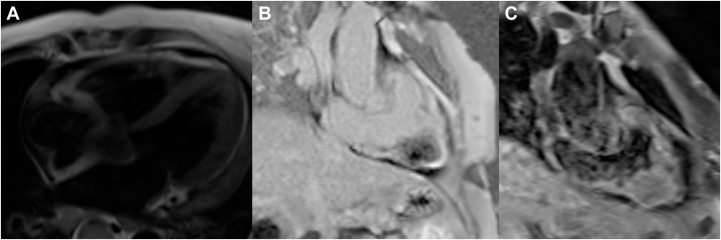
Cardiac Magnetic Resonance Imaging Revealing Right Ventricular Mass Concerning for Metastasis Cardiac magnetic resonance imaging showing phase-sensitive inversion recovery sequence (B) as well as T1 sequence (C) with isointense mass and T2 sequence (A) with hypointense mass within the right ventricle mid to apex measuring 5.1 × 3.7 × 3.5 cm with minimal enhancement and no significant diffusion restriction. These findings favor right intraventricular metastasis with central necrosis.

**Figure 3:**
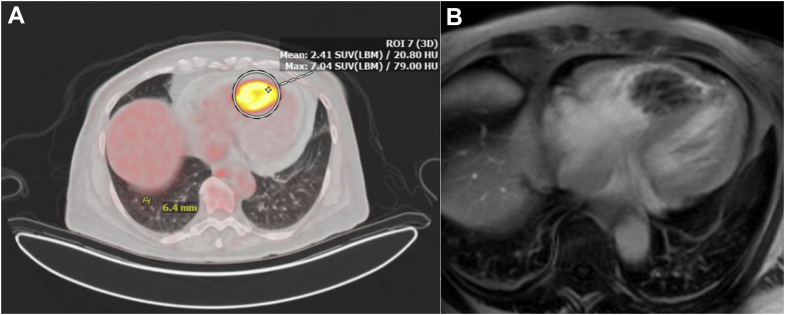
Imaging Demonstrating Right Ventricular Mass (A) Positron emission tomography–computed tomography and (B) cardiac magnetic resonance imaging showing cardiac mass measuring 2.9 × 4.4 × 3.1 cm with central photopenia and maximum standardized uptake value (SUV) of 7.0. These imaging findings are most consistent with a necrotic right ventricular cardiac metastasis. ROI = region of interest.

## Management

Additional workup was guided by a multidisciplinary approach to best determine further management. Interventional cardiology performed a biopsy, and the results were consistent with metastatic squamous cell carcinoma (Figure 4). Cardiothoracic surgery was consulted and deemed that the risks of surgical resection of the mass outweighed the benefits. The oncology team evaluated the patient and started her on the third cycle of chemotherapy with carboplatin and paclitaxel, planning to administer bevacizumab and pembrolizumab as an outpatient.

**Figure 4:**
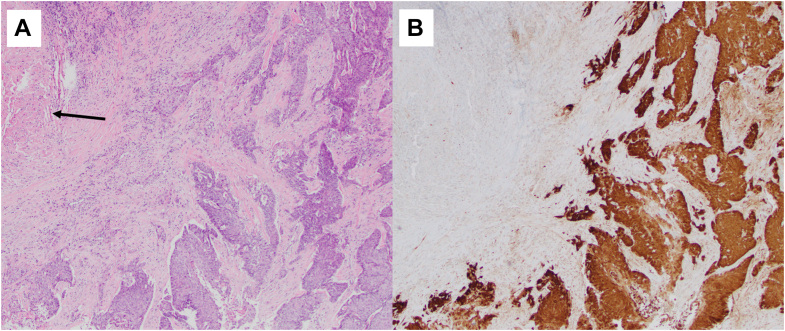
Right Ventricular Mass Biopsy Consistent with Cardiac Metastasis The right ventricular tumor (A) showed squamous cell carcinoma involving the cardiac muscle. The residual cardiac muscle is shown in the upper left by the arrow. (B) The corresponding section of (A) with diffuse p16 positivity in the squamous cell carcinoma. The residual cardiac muscle was negative.

## Outcome and Follow-Up

Given the onset of arrhythmias likely due to interference of conduction pathways by the mass, the patient was discharged with metoprolol succinate, ambulatory cardiac monitoring, and outpatient cardiology follow-up for further arrhythmia management.

After discharge, the patient continued to see oncology and was treated with carboplatin, paclitaxel, bevacizumab, and pembrolizumab. Routine imaging 5 months later, including PET-CT and CMR imaging, revealed that the cardiac metastasis remained stable in size and did not have any evidence of immune checkpoint inhibitor–induced myocarditis. At cardiology follow-up, the patient had a heart monitor for 1 week that revealed 1 episode of NSVT lasting 3 beats with a premature ventricular contraction burden of less than 1%. She continued to take metoprolol succinate without the need for additional interventions given the low arrhythmia burden and symptomatic stability on chemo-immunotherapy.

## Discussion

Cardiac metastasis from cervical cancer is an exceedingly rare phenomenon. The presence of cardiac metastasis has a reported incidence of 1.2% and is most commonly associated with lung cancer, lymphoma, breast cancer, melanoma, and esophageal cancer.^1^^,^^2^ There are infrequent reports of cardiac metastasis involving cancers that originate below the diaphragm, such as cervical cancer.^2^ The presence of cardiac metastasis confers a poor prognosis, with survival ranging from a few months to a maximum of 2 years.^2^ There are multiple routes for metastasis, including direct invasion of adjacent organs, transvenous extension, lymphatic dissemination, and hematogenous dissemination.^2^^,^^3^ Pericardial metastasis is most frequent, followed by epicardial and myocardial involvement with endocardial and intracavitary metastasis accounting for only 3% to 5% of cases.^2^^,^^3^

Our patient metastasized to the right ventricle, and previous cases have found that metastasis typically occurs in the right-sided chambers due to slower velocities conducive to tumor tissue colonization and the lack of filtration through the pulmonary vasculature.^2^ Based on previous reports, more than 80% of cardiac metastasis in cervical cancer occurs within the first 2 years after initial treatment, suggesting these 2 years are a critical period during which patients should be closely monitored for symptoms of cardiac involvement.^4^ In this case, our patient developed metastasis within the first couple of months after initial diagnosis, supporting the need for frequent imaging and follow-up. Interestingly, our patient's PET scan that noted the cardiac metastasis also noted reduction in size of the primary tumor, indicating progression to stage IV disease despite response to chemotherapy.

Echocardiogram is the first-line imaging modality to evaluate cardiac masses due to its widespread availability, lack of contrast or radiation exposure, and dynamic assessment of the cardiac mass relative to surrounding cardiac structures.^3^ However, there remain several limitations to echocardiogram evaluation, including poor acoustic windows and limited assessment of soft tissue characteristics. To obtain better tissue characterization and detect potential metastasis, cardiac CT or CMR is often used.^3^ CMR is often preferred over cardiac CT given its superior soft tissue characterization, multiplanar imaging capabilities, high temporal resolution, ability to assess the degree of vascularity of the mass, and unrestricted field of view.^3^ However, limitations include inadequate assessment of coronary arteries and small mobile masses, as well as the inability to image patients with implanted magnetic devices.^3^ Cardiac CT is an alternative imaging modality, particularly for patients with contraindications to CMR, calcified masses, or masses involving the coronary arteries.^3^ It is also useful in detecting metastases when coupled with ^18^F-fluorodeoxyglucose PET, as metastatic tumors or primary malignant cardiac tumors show significantly higher glucose uptake as quantified by the standardized uptake value as compared with benign cardiac tumors.^3^

Patients with cardiac metastasis may be asymptomatic or present with nonspecific cardiac symptoms that predominantly consist of chest pain, shortness of breath, atrial arrhythmias, heart failure, mechanical obstruction, and cardiac tamponade.^5^ However, ventricular arrhythmias resulting from cardiac metastasis as found in our patient are infrequently described. Although the exact mechanism remains unknown, it has been hypothesized to be due to the formation of a reentrant circuit in the myocardium around or within the tumor.^5^ The management of such arrhythmias is complicated because of the lack of established guidelines, but current practice consists of antiarrhythmic drugs as the first step in management.^5^ Implantable cardioverter-defibrillators are typically not first-line therapy given the risk of electrical storm in cases with a high burden of ventricular arrhythmias and the short life expectancy in these patients.^6^^,^^7^ Implantable cardioverter-defibrillators may be an option for patients whose tumor responds well to treatment but continue to have arrhythmias due to cardiac scar formation. However, this is rarely seen in patients with cardiac metastasis, thus arrhythmia management is largely limited to a pharmacologic approach.^7^ For cases refractory to antiarrhythmic drugs, there are sparse reports of radiofrequency ablation, which may require a dual epicardial and endocardial approach depending on the extent of myocardial involvement.^6^

Management of cardiac metastases should involve a multidisciplinary approach to determine surgical candidacy and appropriate cytotoxic therapy. The decision of whether or not to proceed with surgery is multifactorial and requires consideration of clinical presentation, anatomical feasibility, prognosis, and the patient's goals of care.^8^ Patients who present with hemodynamic instability or ventricular dysfunction due to obstruction caused by the mass may warrant urgent surgical intervention.^8^ However, in stable patients surgery is often not considered first-line management because of its inability to be curative given the systemic metastasis at this stage in the disease.^9^ Elective surgery is typically reserved for patients with excellent prognosis and good functional capacity.^10^ Alternatively, patients with poor prognosis also can be considered for surgery for palliative reasons depending on their clinical symptoms.^9^ In general, given the high risk associated with these surgeries and the short life expectancy at this stage of disease, most patients are managed with nonsurgical options mainly consisting of chemotherapy and symptom management.^7^^,^^9^^,^^10^

## Conclusions

Given the rarity of cardiac metastasis in cervical cancer, optimal management is poorly defined and must consider the patient's life expectancy and functional status. Surgery is reserved for a small subset of patients whereas most patients are managed with chemotherapy and symptom treatment. Patients can suffer from an array of complications, but ventricular arrhythmias are a rarely described phenomenon and lack specific management guidelines. Previous cases have comprised pharmacological therapy with escalation to radiofrequency ablation if required. Our patient developed NSVT due to cardiac metastasis for which she was treated with chemotherapy and a beta blocker. The overall management of these cases requires a multidisciplinary approach that must first consider nonsurgical interventions then escalate to more invasive measures if required.

## Funding Support and Author Disclosures

The authors have reported that they have no relationships relevant to the contents of this paper to disclose.
